# Supplementary material for: AMRomics: a scalable workflow to analyze large microbial genome collections
Source: BMC Genomics. 2024 Jul 22;25:709. doi: 10.1186/s12864-024-10620-8 (PMC11264974; doi:10.1186/s12864-024-10620-8)
Supplement: Supplementary file 1 — Supplementary Material 1. [file 12864_2024_10620_MOESM1_ESM.pdf]

Supplementary Table 1. List of bioinformatics tools used by AMRomics pipeline

Tools	Source	Version	Function
		Single sample analysis	
abricate	https://github.com/tseemann/abricate	1.0.1	Mass screening of contigs for antimicrobial and virulence genes[24]
blast	https://ftp.ncbi.nlm.nih.gov/blast/executables/blast+/LATEST/	2.13.0	NCBI blast package for genome alignment[17]
fastp	https://github.com/OpenGene/fastp	0.23.3	Quick FASTQ reads trimming (adapters, length, quality)[4,5]
FastQC	https://github.com/s-andrews/FastQC	0.11.9	Quality control analysis for high throughput sequencing data[1]
Flye	https://github.com/fenderglass/Flye	2.9-b1768	*De novo* genome assembler for long-reads data[12,15]
mash	https://github.com/marbl/Mash	2.3	Fast genome size, (meta)genome distance estimation using MinHash[20]
mlst	https://github.com/tseemann/mlst	2.19.0	Scan contig files against PubMLST[9] typing schemes[23]
MultiQC	https://github.com/ewels/MultiQC	1.10	Aggregate results from bioinformatics analyses across many samples into a single report[7]
Prokka	https://github.com/tseemann/prokka	1.14.6	Rapid prokaryotic genome annotation[25]
samtools	https://github.com/samtools/samtools	1.17	Manipulate NGS alignment data[6]
seqtk	https://github.com/lh3/seqtk	1.4-r122	Processing, downsampling sequences data in FASTA/Q formats[14]
SPAdes	https://github.com/ablab/spades	3.15.5	*De novo* genome assembler for Illumina short-reads data[22,27]
		Pan-genome comparative analysis	
MAFFT	https://github.com/GSLBiotech/mafft	7.520	Align multiple amino acid or nucleotide sequences[10,11]
msa2vcf	https://github.com/connor-lab/msa2vcf		Turn a fasta-format MSA into one vcf per sequence.
IQTREE	https://github.com/iqtree/iqtree2	2.2.2.3	Build phylogenomics by maximum likelihood[18]
FastTree	http://www.microbesonline.org/fasttree/	2.1.11	Build approximately-maximum-likelihood phylogenetic trees from large alignments of nucleotide[21]
Panta	https://github.com/amromics/panta	1.0	Fast bacterial pangenome analysis[13]

Supplementary Table 2. List of bioinformatics databases used by AMRomics pipeline

Database	Source	Description
PubMLST	https://pubmlst.org	MLST typing scheme for bacterial strains[9]
AMRFinderPlus	https://ftp.ncbi.nlm.nih.gov/pathogen/Antimicrobial_resistance/AMRFinderPlus/database/latest/	Database of antimicrobial resistance genes and point mutations in the assemblies[8]
VFDB	http://www.mgc.ac.cn/VFs/Down/VFDB_setA_nt.fas.gz	Virulence factor database[3,16]
PlasmidFinder	https://bitbucket.org/genomicepidemiology/plasmidfinder_db	Database of origin of replicon sequences to identify plasmid[2]
INTEGRALL	http://integrall.bio.ua.pt/	Database and search engine for integrons, integrases and gene cassette[19]
IntFinder	https://bitbucket.org/genomicepidemiology/intfinder_db	Integron database following INTEGRALL nomenclature[26]

References

1. S. Andrews. FastQC: A Quality Control Tool for High Throughput Sequence Data [Online]. 2010.

2. A. Carattoli, E. Zankari, A. García-Fernández, M. Voldby Larsen, O. Lund, L. Villa, F. Møller Aarestrup, and H. Hasman. In silico detection and typing of plasmids using plasmidfinder and plasmid multilocus sequence typing. *Antimicrobial agents and chemotherapy*, 58(7):3895–3903, 2014.

3. L. Chen, J. Yang, J. Yu, Z. Yao, L. Sun, Y. Shen, and Q. Jin. VFDB: a reference database for bacterial virulence factors. *Nucleic acids research*, 33(suppl_1):D325–D328, 2005.

4. S. Chen. Ultrafast one-pass fastq data preprocessing, quality control, and deduplication using fastp. *iMeta*, page e107, 2023.

5. S. Chen, Y. Zhou, Y. Chen, and J. Gu. fastp: an ultra-fast all-in-one fastq preprocessor. *Bioinformatics*, 34(17):i884–i890, 2018.

6. P. Danecek, J. K. Bonfield, J. Liddle, J. Marshall, V. Ohan, M. O. Pollard, A. Whitwham, T. Keane, S. A. McCarthy, R. M. Davies, and H. Li. Twelve years of SAMtools and BCFtools. *GigaScience*, 10(2), 02 2021. giab008.

7. P. Ewels, M. Magnusson, S. Lundin, and M. Käller. MultiQC: summarize analysis results for multiple tools and samples in a single report. *Bioinformatics*, 32(19):3047, 2016.

8. M. Feldgarden, V. Brover, N. Gonzalez-Escalona, J. G. Frye, J. Haendiges, D. H. Haft, M. Hoffmann, J. B. Pettengill, A. B. Prasad, G. E. Tillman, et al. AMRFinderPlus and the reference gene catalog facilitate examination of the genomic links among antimicrobial resistance, stress response, and virulence. *Scientific reports*, 11(1):1–9, 2021.

9. K. A. Jolley and M. C. Maiden. BIGSdb: scalable analysis of bacterial genome variation at the population level. *BMC bioinformatics*, 11:1–11, 2010.

10. K. Katoh, G. Asimenos, and H. Toh. Multiple alignment of dna sequences with mafft. *Bioinformatics for DNA sequence analysis*, pages 39–64, 2009.

11. K. Katoh and M. C. Frith. Adding unaligned sequences into an existing alignment using mafft and last. *Bioinformatics*, 28(23):3144–3146, 2012.

12. M. Kolmogorov, J. Yuan, Y. Lin, and P. A. Pevzner. Assembly of long, error-prone reads using repeat graphs. *Nature biotechnology*, 37(5):540–546, 2019.

13. D. Q. Le, T. A. Nguyen, T. T. Nguyen, S. H. Nguyen, V. H. Do, C. H. Nguyen, H. T. Phung, T. H. Ho, V. S. Nam, T. Nguyen, H. A. Nguyen, and M. D. Cao. PanTA : An ultra-fast method for constructing large and growing microbial pangenomes. *bioRxiv*, pages 1–9, 2023.

14. H. Li. Github `https://github.com/lh3/seqtk`. 2011.

15. Y. Lin, J. Yuan, M. Kolmogorov, M. W. Shen, M. Chaisson, and P. A. Pevzner. Assembly of long error-prone reads using de bruijn graphs. *Proceedings of the National Academy of Sciences*, 113(52):E8396–E8405, 2016.

16. B. Liu, D. Zheng, S. Zhou, L. Chen, and J. Yang. Vfdb 2022: a general classification scheme for bacterial virulence factors. *Nucleic acids research*, 50(D1):D912–D917, 2022.

17. S. McGinnis and T. L. Madden. BLAST: at the core of a powerful and diverse set of sequence analysis tools. *Nucleic acids research*, 32(suppl_2):W20–W25, 2004.

18. B. Q. Minh, H. A. Schmidt, O. Chernomor, D. Schrempf, M. D. Woodhams, A. Von Haeseler, and R. Lanfear. IQ-TREE 2: new models and efficient methods for phylogenetic inference in the genomic era. *Molecular biology and evolution*, 37(5):1530–1534, 2020.

19. A. Moura, M. Soares, C. Pereira, N. Leitão, I. Henriques, and A. Correia. INTEGRALL: a database and search engine for integrons, integrases and gene cassettes. *Bioinformatics*, 25(8):1096–1098, 2009.

20. B. D. Ondov, T. J. Treangen, P. Melsted, A. B. Mallonee, N. H. Bergman, S. Koren, and A. M. Phillippy. Mash: fast genome and metagenome distance estimation using minhash. *Genome biology*, 17(1):1–14, 2016.

21. M. N. Price, P. S. Dehal, and A. P. Arkin. FastTree 2 – Approximately Maximum-Likelihood Trees for Large Alignments. *PLoS ONE*, 5(3):e9490, mar 2010.

22. A. D. Prjibelski, I. Vasilinetc, A. Bankevich, A. Gurevich, T. Krivosheeva, S. Nurk, S. Pham, A. Korobeynikov, A. Lapidus, and P. A. Pevzner. ExSPAnder: a universal repeat resolver for DNA fragment assembly. *Bioinformatics*, 30(12):i293–i301, 06 2014.

23. T. Seeman. Github `https://github.com/tseemann/mlst`. 2014.

24. T. Seeman. Github `https://github.com/tseemann/abricate`. 2017.

25. T. Seemann. Prokka: rapid prokaryotic genome annotation. *Bioinformatics*, 30(14):2068–2069, 2014.

26. L. Torres-Elizalde, D. Ortega-Paredes, K. Loaiza, E. Fernández-Moreira, and M. Larrea-Álvarez. In silico detection of antimicrobial resistance integrons in salmonella enterica isolates from countries of the andean community. *Antibiotics*, 10(11):1388, 2021.

27. I. Vasilinetc, A. D. Prjibelski, A. Gurevich, A. Korobeynikov, and P. A. Pevzner. Assembling short reads from jumping libraries with large insert sizes. *Bioinformatics*, 31(20):3262–3268, 06 2015.
